# Motor Pathways Reorganization following Surgical Decompression for Degenerative Cervical Myelopathy: A Combined Navigated Transcranial Magnetic Stimulation and Clinical Outcome Study

**DOI:** 10.3390/brainsci14020124

**Published:** 2024-01-25

**Authors:** Alessandro Boaro, Sonia Nunes, Chiara Bagattini, Valeria Di Caro, Francesca Siddi, Fabio Moscolo, Christian Soda, Francesco Sala

**Affiliations:** 1 Section of Neurosurgery, Department of Neurosciences, Biomedicine and Movement Sciences, University of Verona, 37124 Verona, Italy; alessandro.boaro@univr.it (A.B.); sonia@iomneuro.com (S.N.); chiara.bagattini@univr.it (C.B.); valeria.dicaro@univr.it (V.D.C.); francesca.siddi@univr.it (F.S.); 2 Neurosurgery Unit, Carlo Poma Hospital, 46100 Mantova, Italy; dottmoscolo@gmail.com; 3 Institute of Neurosurgery, Azienda Ospedaliera Universitaria Integrata, 37126 Verona, Italy; christian.soda@aovr.veneto.it

**Keywords:** degenerative cervical myelopathy, nTMS, plasticity, corticospinal reorganization

## Abstract

(1) Background: Degenerative cervical myelopathy is one of the main causes of disability in the elderly. The treatment of choice in patients with clear symptomatology and radiological correlation is surgical decompression. The application of navigated transcranial magnetic stimulation (nTMS) techniques has the potential to provide additional insights into the cortical and corticospinal behavior of the myelopathic cord and to better characterize the possible extent of clinical recovery. The objective of our study was to use nTMS to evaluate the effect of surgical decompression on neurophysiological properties at the cortical and corticospinal level and to better characterize the extent of possible clinical recovery. (2) Methods: We conducted a longitudinal study in which we assessed and compared nTMS neurophysiological indexes and clinical parameters (modified Japanese Orthopedic Association score and nine-hole pegboard test) before surgery, at 6 months, and at 12 months’ follow-up in a population of 15 patients. (3) Results: We found a significant reduction in resting motor threshold (RMT; average 7%), cortical silent period (CSP; average 15%), and motor area (average 25%) at both 6 months and 12 months. A statistically significant linear correlation emerged between recruitment curve (RC) values obtained at follow-up appointments and at baseline (r = 0.95 at 6 months, r = 0.98 at 12 months). A concomitant improvement in the mJOA score and in the nine-hole pegboard task was observed after surgery. (4) Conclusions: Our results suggest that surgical decompression of the myelopathic spinal cord improves the neurophysiological balance at the cortical and corticospinal level, resulting in clinically significant recovery. Such findings contribute to the existing evidence characterizing the brain and the spinal cord as a dynamic system capable of functional and reversible plasticity and provide useful clinical insights to be used for patient counseling.

## 1. Introduction

Degenerative cervical myelopathy is one of the main causes of disability in the elderly. The treatment of this condition is not straightforward, as it can entail a more conservative approach, which mainly translates into physiotherapy and pain control, or a more aggressive approach consisting of surgical operation for the decompression of neural structures [1]. The decision to undergo surgical treatment is mostly based on the evaluation of the clinical picture and magnetic resonance imaging (MRI) findings. However, even considering the continuous advancement in the understanding and evaluation of medical imaging [2,3] as well as the wide spectrum of operative approaches available [4,5], there is still uncertainty as to the capability of physicians to provide a reliable prospect of the possible gain in terms of functional recovery based only on these two elements. The neurophysiological study of patients affected by acute or chronic spinal cord injury provided additional clarity on the mechanisms underlying the impairment of spinal cord functions [6,7]. In particular, recent developments and the application of navigated transcranial magnetic stimulation (nTMS) provided key insights into the cortical and corticospinal behavior of myelopathic patients compared to healthy subjects [8,9,10,11]. Current evidence suggests the presence of a functional reserve capacity of the brain that is employed during the chronic development of cervical myelopathy and that tends to be exhausted with the worsening and prolongation of the condition [10]. We conducted a longitudinal study in order to evaluate the effect of surgical decompression of the myelopathic cervical cord on cortical and corticospinal neurophysiological properties and therefore to better characterize the extent of possible clinical recovery. We evaluated a group of patients by means of nTMS in the pre-operative and post-operative phases, under the hypothesis that surgical decompression of the spinal cord would lead to an improvement in both neurophysiological and clinical parameters, hence avoiding the exhaustion of the functional reserve of the brain.

## 2. Materials and Methods

### 2.1. Patient Recruitment

Patient recruitment was conducted at the Neurosurgery Department of Verona University Hospital between August 2021 and November 2022. Inclusion criteria were (a) clinical signs of cervical myelopathy, such as gait dysfunction, hand impairment, and/or the presence of long tract signs; (b) signs of radiologically confirmed cervical myelopathy and/or moderate to severe cervical stenosis; (c) indication for surgical decompression; (d) age above 18 years; (e) absence of pacemaker, deep brain stimulation electrodes, or pregnancy (TMS-related safety reasons). Patients with acute spinal cord injury or patients with a prior history of surgically treated cervical myelopathy were not included in this study. All patients underwent the gold-standard peri-operative evaluations needed to pursue standard-of-care practices. Dedicated informed consent was obtained, signed, and stored.

### 2.2. nTMS Parameter Determination

All patients underwent nTMS brain mapping (STM9000; EBNeuro, Florence, Italy) using a 70 mm figure-of-eight coil combined to a neuronavigation system (NetBrain 9000; EBNeuro) which allowed for the upload of patient MRI and produced high-quality brain cortex reconstruction. Specifically, volumetric T1 sequences without contrast were used, given the superior results obtained for surface reconstruction compared to the other sequences. The first digital interosseous (FDI) muscle was used as the target muscle (except in two cases in which abductor pollicis brevis had to be used due to advanced muscle wasting) and extensor communis digitorum was used as the control muscle. 

Surface electrodes were preferably used, but in two cases, due to difficulties in obtaining viable MEPs, needle electrodes had to be placed. The following parameters were obtained: resting motor threshold (RMT), recruitment curve (RC), cortical silent period (CSP), motor area extension. All the parameters were obtained stimulating both the left and the right hemisphere (recording from the right and the left target muscles, respectively). 

#### 2.2.1. Resting Motor Threshold

The resting motor threshold (RMT) for the target muscle was obtained as a measure of cortical excitability. The hotspot was identified as explained in previous works [12], and the RMT was defined as the lowest output intensity producing at least 5 motor evoked potentials (MEPs) ≥ 50 μV (peak to peak) out of 10 consecutive trials.

#### 2.2.2. Recruitment Curve

To assess corticospinal excitability, the recruitment curve (RC) protocol was used. The target muscle’s hotspot was stimulated at varying intensities between 80% and 120% of the RMT at steps of 10%. The resulting MEP amplitudes were plotted against the corresponding stimulation intensities.

#### 2.2.3. Cortical Silent Period

Cortical and corticospinal inhibition were assessed through the cortical silent period (CSP). It was obtained by having the patient to maintain a motor contraction of the target muscle in a continuous manner, whilst a set of ten trials of hotspot simulation at 120% RMT were delivered. To estimate the relative CSP, the time occurring from the end of the MEP to the restart of electrical activity was measured. The average value of the ten trials was calculated to obtain the CSP. 

#### 2.2.4. Motor Area Extension

Once the previous parameters were obtained, the coil was used to define the target muscle’s motor area by mapping at 110% RMT. Viable MEPs with peak-to-peak value of at least 50 μV were included. The set of points obtained in the 3D reconstructed cortex was then projected onto a 2D surface by means of a medical imaging editor software (3D Slicer 5.4.0, Boston, MA, USA) and the resulting area was calculated.

### 2.3. Evaluation of Neurological Status

Each patient was evaluated using a combination of a clinical score and a dexterity test. In detail, the clinical score used was the modified Japanese Orthopedic Association (mJOA) score, which reflects disability at the level of the upper and lower extremities as well as sensory and bladder functions [13]. The dexterity test used was the 9-hole pegboard test, which consists in timing the task of putting 9 pins in a 3 × 3 board and subsequently removing them one by one. The test was conducted on each hand separately. None of the patients were undergoing specific physiotherapy programs for muscle strengthening at the time of enrolment.

### 2.4. Pre-Operative and Follow-Up Evaluation Protocol

Each patient underwent clinical evaluation (via mJOA and 9-hole pegboard test) and nTMS protocol a total of three times. The first session was scheduled within two weeks preceding the surgery in order to provide a clinical and neurophysiological baseline (pre-op). The following sessions were scheduled at 6 (6 mo fu) and 12 months after surgery (12 mo fu). Before the first session, each patient underwent a brain MRI without contrast in order to obtain the volumetric T1 sequence for navigation. The same cortical surface reconstruction was used across all sessions. With regards to the nTMS protocol, follow-up appointments were conducted by the same investigator, who did not review the results of previous appointments in order to avoid potential biases. The data obtained across all the sessions were digitized in an anonymized way (inclusive of mJOA and 9-hole pegboard test results) and stored within secure university servers.

### 2.5. Data Analysis 

Neurophysiological and clinical data for all patients were put in a dedicated Excel 16.81 worksheet in order to perform data analysis. The analysis was conducted at a population level, combining the left and right hemispheres, with data expressed as mean ± standard deviation (SD). We employed Student’s *t*-test for paired samples to compare the neurophysiological and clinical parameters obtained at 6 months’ and 12 months’ follow-up sessions with the values obtained at baseline. The comparisons were performed both considering the absolute values of the parameters as well as expressed as the percentage of difference with respect to the baseline (where baseline values constituted the 100%). The *p* value obtained was assessed in the context of a two-tailed test with α set at 0.05. In addition, we tested the presence of a linear correlation between the baseline and follow-up sessions with regards to recruitment curves using the Pearson correlation coefficient with α set at 0.05.

## 3. Results

### 3.1. Patient Population

**Figure 1:**
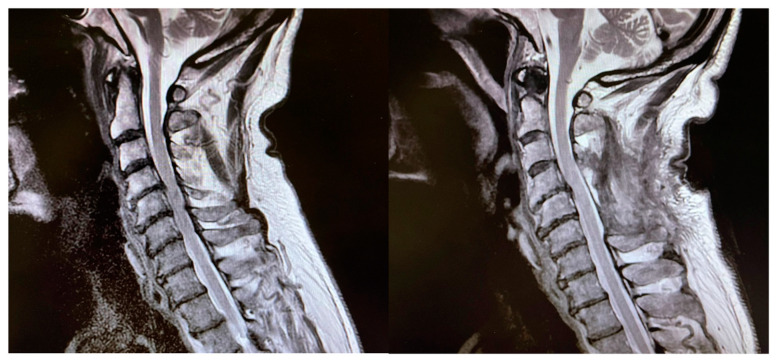
On the left, pre-operative T2 MRI image showing spinal cord compression due to degenerative cervical spondylosis; on the right, post-operative T2 MRI image showing satisfactory decompression of the spinal cord after multi-level laminectomy.

A total of 15 patients were included in the study. The patient population presented a mean age of 60 ± 9.1 years, the majority were men (4:1) and 14 out of 15 patients presented with degenerative cervical myelopathy, while one patient presented with chronic myelopathy due to progressive growth of a cervical neuroma. Ten patients out of fifteen underwent a posterior approach for either laminectomy, open-door laminoplasty, or laminotomy for tumor resection; the remaining five underwent anterior cervical discectomy and fusion (Table 1, Figure 1).

### 3.2. nTMS Parameters and Motor Area Extension

#### 3.2.1. Resting Motor Threshold

RMT measurements at baseline presented mean and standard deviation values of 64.1 ± 14.1 for the left side, 65.1 ± 12.4 for the right side, and 64.6 ± 12.9 for both sides combined. At 6 months’ follow-up, the RMT was 60.2 ± 14.3 for the left side, with a decrease of 6.6% on average, 62.4 ± 13.9 for the right side, with a decrease of 7% on average, and an overall RMT value of 61.23 ± 13.7, with a decrease of 7% on average. At 12 months’ follow-up, the RMT was 62.3 ± 14.1 for the left side, with a decrease of 7% on average, 61.2 ± 12.9 for the right side, with a decrease of 7% on average, and the overall RMT value was 61.7 ± 13.1, with a decrease of 6.8% on average. The comparison between follow-up and baseline values showed a significant decrease at both 6 and 12 months and in both absolute and percentage values. The comparison between the two follow-up sessions did not reach statistical significance (Table 2).

#### 3.2.2. Recruitment Curve

**Figure 2:**
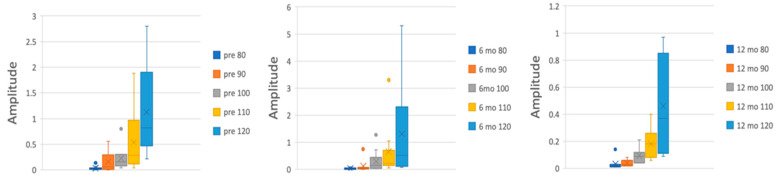
RC trends at baseline, 6 months’ follow-up, and 12 months’ follow-up.

MEP amplitudes showed a general progressive increase in response values as the stimulation intensities increased. This trend was more evident in baseline values and at 6 months’ follow-up compared to 12 months’ follow-up. Specifically, the values for the left and right sides combined at baseline ranged between an average of 0.09 mV at 80%RMT and an average of 1.03 mV at 120%RMT; at 6 months, the values ranged between an average of 0.03 mV at 80%RMT and an average of 1.07 mV at 120%RMT; finally, at 12 months, the values ranged between an average of 0.03 mV at 80%RMT and an average of 0.4 mV at 120%RMT. We found a statistically significant linear correlation between the values obtained at follow-up sessions and the ones obtained at baseline (r = 0.95 for the 6-month values, r = 0.98 for the 12-month values) (Table 3 and Table 4, Figure 2).

#### 3.2.3. Cortical Silent Period

CSP measurements at baseline presented mean and standard deviation values of 140.4 ± 35.4 for the left side, 169.2 ± 37.3 for the right side, and 154 ± 38.2 for both sides combined. At 6 months’ follow-up, the CSP was 125.4 ± 44.4 for the left side, with a decrease of 11.4% on average, 137.1 ± 40.8 for the right side, with a decrease of 21.4% on average, and an overall CSP value of 130.9 ± 41.8, with a decrease of 16.1% on average. At 12 months’ follow-up, the CSP was 123.1 ± 26.9 for the left side, with a decrease of 6.2% on average, 122.6 ± 27.6 for the right side, with a decrease of 20.2% on average, and an overall CSP value of 122.9 ± 22.7, with a decrease of 12.6% on average. The comparison between follow-up and baseline values indicated a significant decrease at both 6 and 12 months and in both absolute and percentage values. The comparison between the two follow-up sessions did not reach statistical significance (Table 2).

#### 3.2.4. Motor Area Extension 

Motor area measurements (in cm^2^) at baseline presented mean and standard deviation values of 6.8 ± 2.9 for the left side, 6 ± 4.3 for the right side, and 6.6 ± 2.8 for both sides combined. At 6 months’ follow-up, the motor area was 5.1 ± 2.7 for the left side, with a decrease of 20% on average, 4.8 ± 2.2 for the right side, with a decrease of 22.7% on average, and an overall motor area value of 5 ± 2.4, with a decrease of 21.2% on average. At 12 months’ follow-up, the motor area was 3.3 ± 1.8 for the left side, with a decrease of 41% on average, 3.8 ± 0.8 for the right side, with a decrease of 18.8% on average, and an overall motor area value of 3.5 ± 1.4, with a decrease of 30.8% on average. The comparison between follow-up and baseline values showed a significant decrease at both 6 and 12 months and in both absolute and percentage values. The comparison between the two follow-up sessions did not reach statistical significance (Table 2).

### 3.3. Clinical Evaluation

Clinical evaluation consisted in calculating the mJOA score and assessing bilateral hand dexterity with the nine-hole pegboard test. With regards to mJOA, we observed a pre-operative average value of 12.8, which characterized the patients as affected by cervical myelopathy of mild-to-moderate relevance. After surgery, we noted an improvement in the mJOA score of roughly 3 points at both 6 months (average 16) and at 12 months (average 15.6), which categorized the patients as affected by mild to no symptoms (Table 1 and Table 5). With regards to the dexterity test, at baseline, patients were able to complete the task in 33.6 ± 13.2 s overall; at follow-up appointments, the time needed by patients to complete the task was significantly reduced, by an average of 8 s, which was evident in the left and right side combined as well as when considered separately (Table 1 and Table 5).

## 4. Discussion

In our study, we were able to investigate, by means of nTMS, neurophysiological changes at the cortical and corticospinal level induced by surgical decompression in patients affected by cervical myelopathy. These changes consisted in both spatial and electro-chemical adaptations of the motor pathways and corresponded to a clinically significant improvement. The removal of the compression forces acting on the cervical cord showed evidence of functional reorganization of the motor pathways; in particular, of a reduction in the inhibition status of the brain (reduced CSP), an improved excitability of cortical connections (reduced RMT), and a reduction in the area responsible for motor activation of the target muscle (reduction in motor area extension).

RMT reflects the excitability of connections occurring at the corticocortical level and the related corticospinal connections. An elevation in the RMT has been observed in patients affected by cortical atrophy. In such situations, where a reduced number of neurons and connections are available, a higher stimulation intensity is needed in order to obtain a viable response. With regards to chronic spinal cord damage, a comparison between healthy subjects and patients with cervical myelopathy has shown that even though the RMT tends to be higher in patients compared to controls, the difference does not reach statistical significance [10]. In our study, where the subjects acted as their own controls, we were able to see a significant reduction in RMT values between the pre-operative baseline and the post-operative follow-up. Such a scenario suggests that while excitability was impaired by the presence of myelopathy and spinal cord compression, these changes are not necessarily permanent, and when the triggering mechanism is removed, the system is able to return to more physiological parameters slowly but steadily. With regards to RC, which reflects both the number of excitable elements at the point of stimulation as well as the integrity of axonal connections to spinal motor neurons [14], it has been observed that in patients with severe clinical symptoms of cervical myelopathy, the RC slope tends to flatten. Such findings suggest that regardless of the increase in stimulation intensity, in case of a sufficiently advanced deterioration of spinal cord fibers, obtaining a viable response becomes increasingly difficult. On the other hand, in the patient cohort in our study, the pre-surgical clinical condition was mild to moderate at most and the comparison between the RC obtained before surgery and at 6 months’ follow-up did not present substantial changes in the curve slope. Such results most likely suggest that patients with a symptomatology which is not yet severe are able to preserve a certain degree of corticospinal excitability. Regarding the CSP, it has been observed that the cortical silent period is mainly due to inhibitory processes involving GABAergic neurons at the cortical level. The presence of a shift towards inhibition has been measured both in the case of acute damage to the spinal cord as well as in pathologies involving cortical and subcortical neural structures like ischemic stroke and multiple sclerosis. In patients with degenerative cervical myelopathy, there is a certain degree of controversy, as results from different works provided evidence of different behaviors of the CSP, which could be shorter, longer, or not significantly different [15,16,17]. In our study, by comparing patients before and after surgery in the setting of a within-subjects design, we were able to eliminate the intrinsic variability that would be present in comparison between different subjects. We found a significant reduction in CSP values at both 6 and 12 months compared to baseline, therefore confirming an inhibitory effect, potentially due to the presence of both myelopathy and active compression forces on the cord. Finally, we noticed a significant decrease in the dimensions of the motor areas of the target muscle at both 6 and 12 months compared to baseline. The area tended to shrink back towards the precentral gyrus. This behavior is in accordance with previous studies showing the expansion of the motor area towards the pre-motor section of the cortex in both animals and human subjects in case of damage to the cortex as well as spinal cord injury [18,19]. As we mostly included patients with mild-to-moderate symptoms, who, according to the theory of the corticospinal reserve capacity, did not exhaust their functional capacity, the resolution of compression to the spinal cord seemed to allow for a reduction in the number of neurons used to serve the target muscle. Patients with severe symptoms of myelopathy and healthy subjects present a motor area of the target muscle that is smaller than that of patient with mild-to-moderate symptoms, suggesting that severe patients have exhausted the additional capacity provided by pre-motor areas, whereas patients with mild-to-moderate symptoms switched off the use of the additional area, most likely as this was no longer required. 

Along with neurophysiological changes, we could discern a parallel change in clinical measures, which showed improvement in both objectively measured hand dexterity and patient-reported functional outcomes. With regards to the clinical picture of patients undergoing surgery for spinal cord decompression for cervical myelopathy, for years, it was believed—and patients were informed about the fact—that the goal of surgery was to stop or slow down the progression of the pathology. Only in recent years, thanks to objective and accurate measures of patient outcomes, it was observed that surgery had the opportunity to provide actual improvement [1,20]. The evidence provided by our study is in line with the recent literature, as we indicated an improvement both in patient-reported scores by means of the mJOA and in the dexterity test in the form of the nine-hole pegboard test. Despite the fact that the mJOA measurement did not show a statistically significant improvement, we have to consider that even a difference of one or two points, while difficult to detect by means of a statistical test, can be considered clinically significant to patients. In this regard, the use of an objective measure for dexterity (i.e., the nine-hole pegboard test) confirmed the improvement in the ability to use fine motor skills in myelopathic patients after surgery. 

The improvement in the excitability–inhibition balance at the cortical level confirmed by the reduction in RMT and CSP values is congruous with the improvement observed in the fine motor skills of the patients. On the other hand, as explained above, whilst we would have expected an improvement in the RC slope after surgery, as suggested by the comparison between myelopathic patients and healthy subjects in a previous work [10], we did not find a significant improvement, which could be due to the mild symptomatology presented by our study population.

It was interesting to observe that all the significant variations in both neurophysiological and clinical parameters occurred over the course of the first 6 months, while no further difference was detected comparing the 12 months’ follow-up with the 6 months’ sessions. Such results suggest that, while patients may subjectively experience a progressive improvement over the course of many months, sometimes up to a year, the net benefit of the surgical operation happens during the first months. 

Overall, the evidence provided by our study suggests that surgical intervention of removing the compression causing chronic damage to the spinal cord has the potential to improve the neurophysiological balance at the cortical and corticospinal level in the form of a shift towards an increased level of excitability, eventually leading to clinical improvement, mostly over the first six months.

Compared to previous studies addressing the neurophysiological evaluation of damage to the spinal cord—both acute and chronic [6,7,10]—our work has merit as the same patients were used as their own controls before and after surgery, contributing to a reduction in physiological inter-subject variability. In addition, the possibility of applying navigational features to the TMS allowed for a more precise definition of motor area boundaries, increasing precision in the evaluation of changes before and after surgery.

In the interpretation of our results, we have to take into consideration some important limitations. Most importantly, our sample size was small and our cohort of patients came from a single neurosurgical unit; such elements may limit the generalization of our findings. In addition, while we tried to schedule follow-up sessions as consistently as possible, a few patients had to be evaluated in the range of ±1 month with regards to the 6 months’ and 12 months’ follow-ups due to personal needs, as some of them did not live nearby. We did not find a specific reason for the gender-related class imbalance. We believe that, in addition to chance alone, the small sample size as well as the stringent inclusion criteria (heart-related problems are more common in men, pregnancy status) may have played a role in skewing the balance. On the other hand, the study conducted has some important strengths, the first being that it was a prospective study in which the study settings were controlled for in all subjects and predetermined by a study protocol that was strictly followed. In addition, the within-subjects design had the significant advantage of allowing for a more precise quantification of the effect of surgery on neurophysiological and clinical parameters. Such an approach, in contrast to comparison between different subjects, allows us to observe and measure what is occurring at a single-patient level whilst at the same time providing the necessary information to conduct statistical analysis at a population level. 

## 5. Conclusions

In summary, the findings of our study contribute to the existing evidence characterizing the brain and the spinal cord as a dynamic system capable of functional and reversible plasticity and provide useful clinical insights to be used during patient counseling. Further research involving higher patient numbers and potentially including additional imaging techniques such as tractography and more advanced settings such as TMS with a robotized arm will have the potential to further refine our findings, allowing for a progressively more personalized approach to patients with degenerative cervical myelopathy. 

## Figures and Tables

**Table 1:** Demographics of patient population and overall measures of clinical recovery. SD: standard deviation, mJOA: modified Japanese Orthopedic Association score, pt: pegboard test.

N. of patients	15
Gender (M/F)	4:1
Mean age (years ± SD)	60 ± 9.1
Disease	
Degenerative	94%
Neoplastic	6%
Surgical approach	
Posterior	66%
Anterior	33%
mJOA average recovery	~3 points
9-hole pt average improvement	8 s

**Table 2:** RMT, CSP, and motor area extension results at baseline and at follow-up sessions. RMT: resting motor threshold; CSP: cortical silent period; mo: months; fu: follow-up; abs: absolute value.

	Sides Combined			*p* Value	Left Side			Right Side		
	Pre-Op	6 mo fu	12 mo fu		Pre-Op	6 mo fu	12 mo fu	Pre-Op	6 mo fu	12 mo fu
RMT (% stim): abs	64.6 ± 12.9	61.23 ± 13.7	61.7 ± 13.1	**<0.05**	64.1 ± 14.1	60.2 ± 14.3	62.3 ± 14.1	65.1 ± 12.4	62.4 ± 13.9	61.2 ± 12.9
CSP (ms): abs	154 ± 38.2	130.9 ± 41.8	122.9 ± 22.7	**<0.05**	140.4 ± 35.4	125.4 ± 44.4	123.1 ± 26.9	169.2 ± 37.3	137.1 ± 40.8	122.6 ± 27.6
motor area (cm^2^): abs	6.6 ± 2.8	5 ± 2.4	3.5 ±1.4	**<0.05**	6.8 ± 2.9	5.1 ± 2.7	3.3 ± 1.8	6± 4.3	4.8 ± 2.2	3.8 ± 0.8
RMT (% stim): %	100	93.18 ± 6.5	93 ± 7	**<0.05**	100	93.4 ± 6.4	93 ± 8.5	100	93 ± 7.1	93 ± 5.6
CSP (ms): %	100	83.91 ± 15	87.4 ± 22	**<0.05** (6 mo fu)	100	88.6 ± 13.7	93.8 ± 25	100	78.6 ± 15.5	79.8 ± 16.8
motor area (cm^2^): %	100	78.8 ± 18.3	69.2 ± 37.7	**<0.05**	100	80 ± 23.2	59 ± 41.7	100	77.3 ± 12.21	81.2 ± 32.3

**Table 3:** Recruitment curve values at baseline and at follow-up appointments.

	Sides Combined			Left Side			Right Side		
	Baseline	6 mo fu	12 mo fu	Baseline	6 mo fu	12 mo fu	Baseline	6 mo fu	12 mo fu
% or RMT									
80	0.09	0.03	0.03	0.0352	0.031	0.0337	0.152	0.036	0.04
90	0.28	0.1	0.06	0.1566	0.118	0.039	0.435	0.09	0.09
100	0.21	0.36	0.1	0.225	0.296	0.094	0.2	0.43	0.12
110	0.54	0.5	0.2	0.536	0.651	0.18	0.54	0.338	0.26
120	1.03	1.07	0.4	1.125	1.31	0.46	0.92	0.77	0.4

**Table 4:** Linear correlation with regards to RCs at baseline and follow-up appointments.

		Pearson r	t Statistic	*p* Value
Combined				
	baseline	-	-	-
	6 mo fu	0.95682823	8.66	**<0.05**
	12 mo fu	0.98650844	4.9	**<0.05**

**Table 5:** Nine-hole pegboard and mJOA results at baseline and at follow-up sessions. mo: months; fu: follow-up; na; not applicable.

	Overall			*p* Value	Left Side			Right Side		
	Pre-Op	6 mo fu	12 mo fu		Pre-Op	6 mo fu	12 mo fu	Pre-Op	6 mo fu	12 mo fu
9-hole pegboard (s)	33.6 ± 13.2	25.9 ± 6	25.3 ± 4.2	**<0.05**	32.4 ± 8.9	25.7 ± 4.7	25 ± 3.2	34.2 ± 17.1	26.2 ± 7.3	25.6 ± 5.2
mJOA	12.8 ± 2.9	16 ± 1.77	15.6 ± 2.5	>0.05	na	na	na	na	na	na

## Data Availability

The data presented in this study are available on request from the corresponding author. The data are not publicly available due to presence of Personally Identifiable Information (PII).

## Abbreviations

nTMS	navigated transcranial magnetic stimulation
FDI	first digital interosseous
RMT	resting motor threshold
RC	recruitment curve
CSP	cortical silent period
mJOA	modified Japanese Orthopedic Association
MEPs	motor evoked potentials
